# Supplementary material for: O-methylated N-glycans Distinguish Mosses from Vascular Plants
Source: Biomolecules. 2022 Jan 15;12(1):136. doi: 10.3390/biom12010136 (PMC8773788; doi:10.3390/biom12010136)
Supplement: Supplementary file 1 [file biomolecules-12-00136-s001.zip › biomolecules-1530299-supplementary.pdf]

Supporting Information for

O-methylated N-glycans Distinguish Mosses from Vascular Plants

David Stenitzer [1], Reka Mocsai [1], Harald Zechmeister [2], Ralf Reski [3], Eva L. Decker [3], Friedrich Altmann [1,*]

[1] Department of Chemistry, Institute of Biochemistry, Universität für Bodenkultur Wien, Muthgasse 18, A-1190 Vienna, Austria; david.stenitzer@boku.ac.at (D.S.); reka.mocsai@boku.ac.at (R.M.); friedrich.altmann@boku.ac.at (F.A.)

[2] Department of Botany and Biodiversity Research, University of Vienna, Rennweg 14, 1030 Vienna, Austria; harald.zechmeister@univie.ac.at (H.Z.).

[3] Plant Biotechnology, Faculty of Biology, University of Freiburg, Schaenzlestrasse 1, 79104 Freiburg, Germany; ralf.reski@biologie.uni-freiburg.de (R.R.); eva.decker@biologie.uni-freiburg.de (E.L.D.).

* Correspondence: friedrich.altmann@boku.ac.at (F.A)

Table S1: **Identification of the 14 Da mass increment as methyl group rather than oxidation.** The measured and calculated mass increments are given. The 0.01 % (p = 0.0001) confidence interval was calculated from the eight values.

Glycan	m/z	delta		Statistics	
MMX	**1065.358**			average	14.020
	1079.380	14.022		std. dev.	0.008
	1093.390	14.010		conf. int 0.01 %	+/- 0.015
MMXF	**1211.422**				
	1225.438	14.016		upper limit	14.035
	1239.466	14.028		lower limit	14.005
MGnX	**1268.446**			of 0.01 %	
	1282.463	14.017		conf.-interval	
	1296.474	14.011			
MGnXF	**1414.498**			calc. increment	
	1428.519	14.021		methyl	14.016
	1442.551	14.032		oxidation	13.979

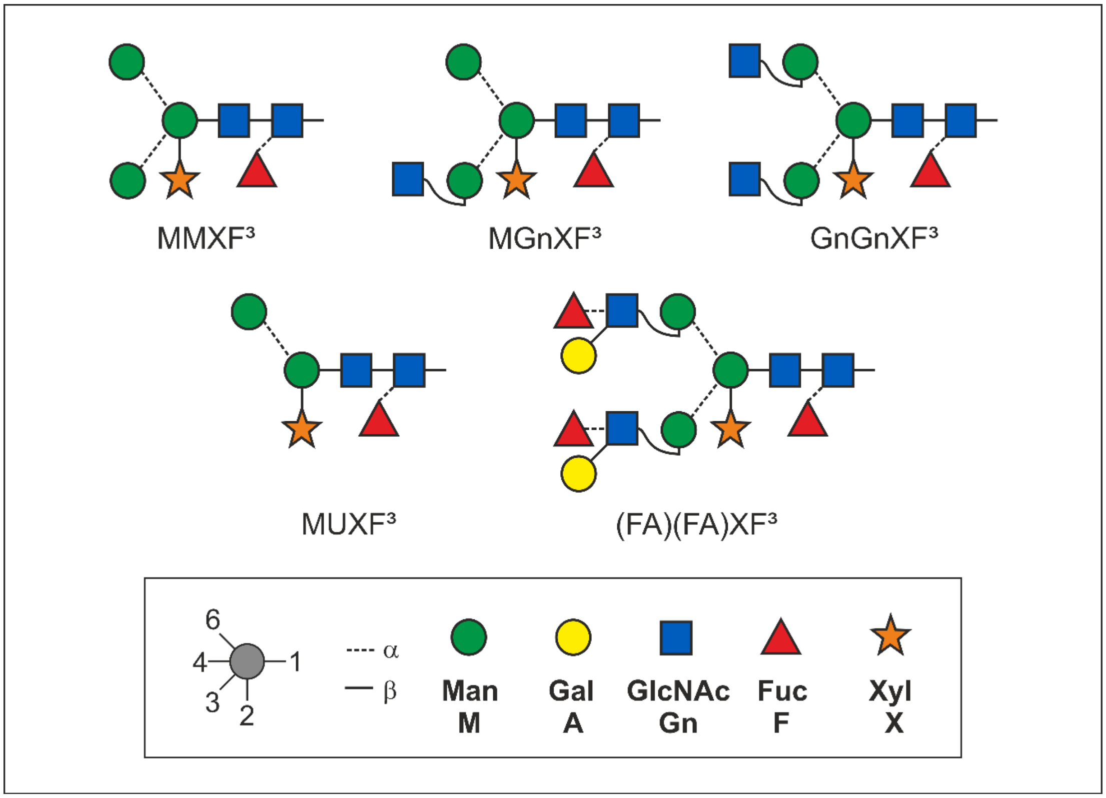

Figure S1: **Structures of plant N-glycans.** The proglycan nomenclature lists the terminal residues in a counter clockwise manner.

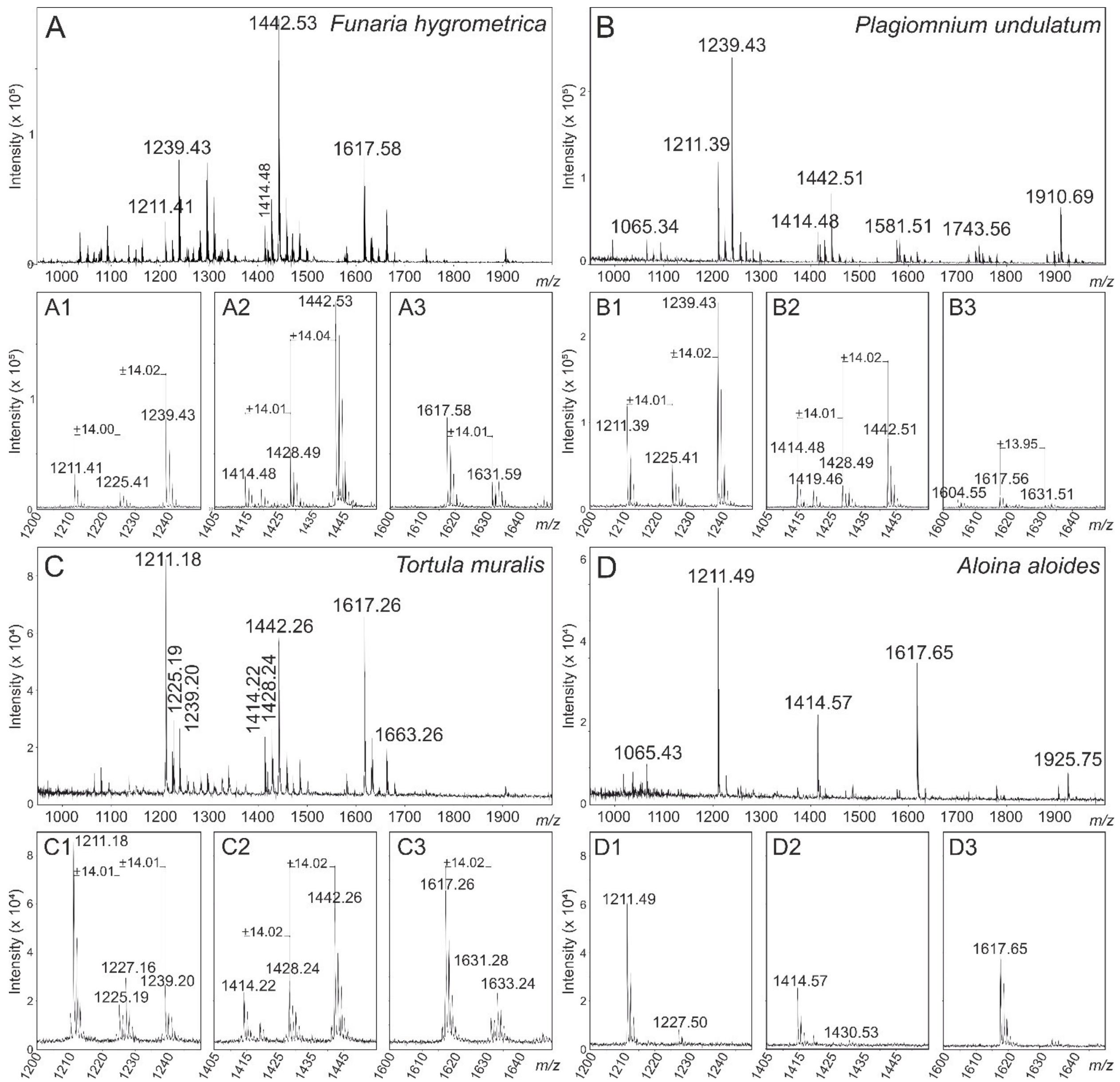

Figure S2: **MALDI-TOF measurements from different mosses.** MALDI-TOF measurements from native N-glycan pools from *Funaria hygrometrica* (**A**), *Plagiomnium undulatum* (**B**), *Tortula muralis* (**C**) and *Aloina aloides* (**D**). A1-D3 show zoomed in areas of the overall spectra.

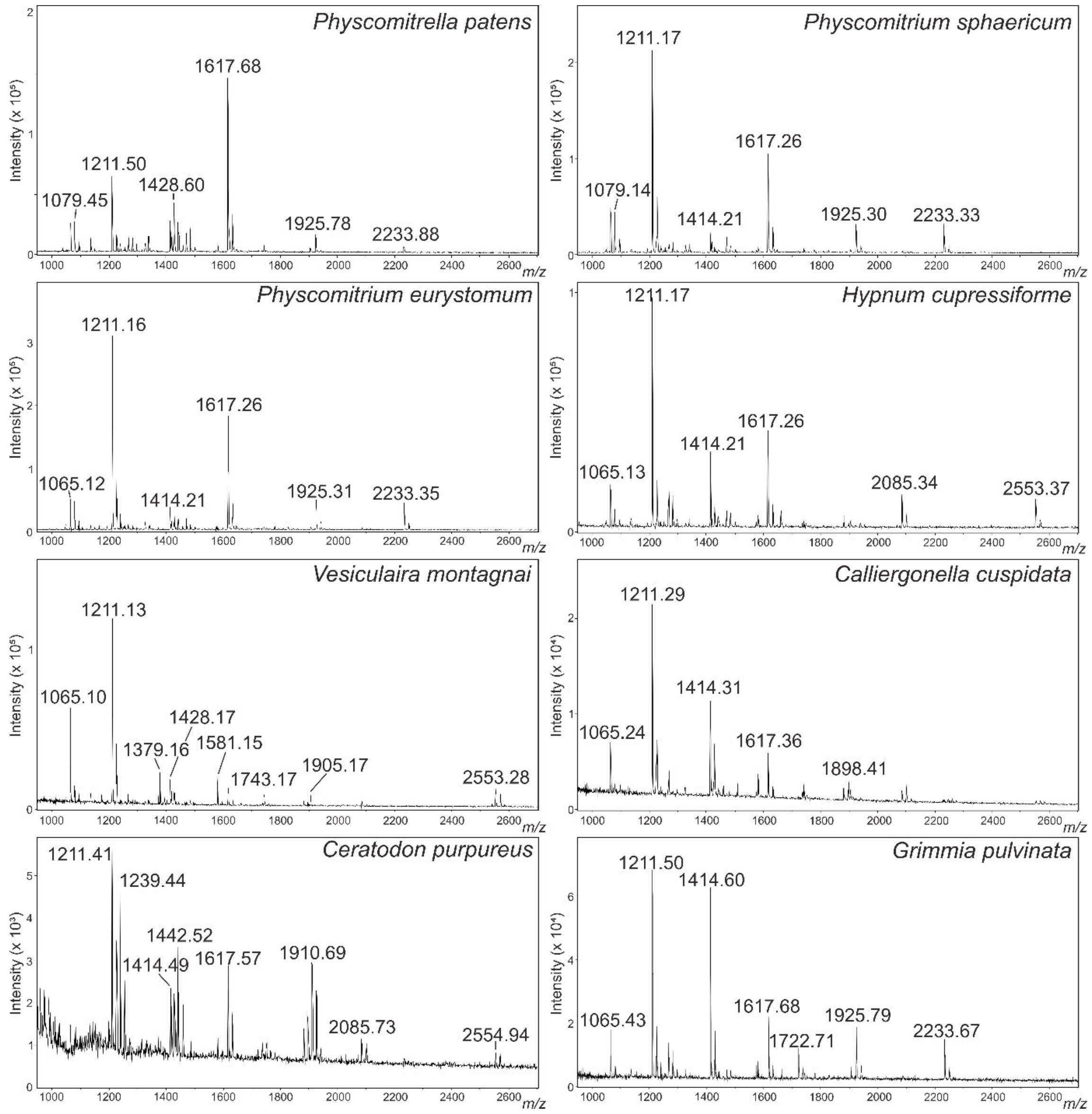

Figure S3A: **MALDI-TOF-MS of different moss species.** Whole glycan pools from the analysed moss species.

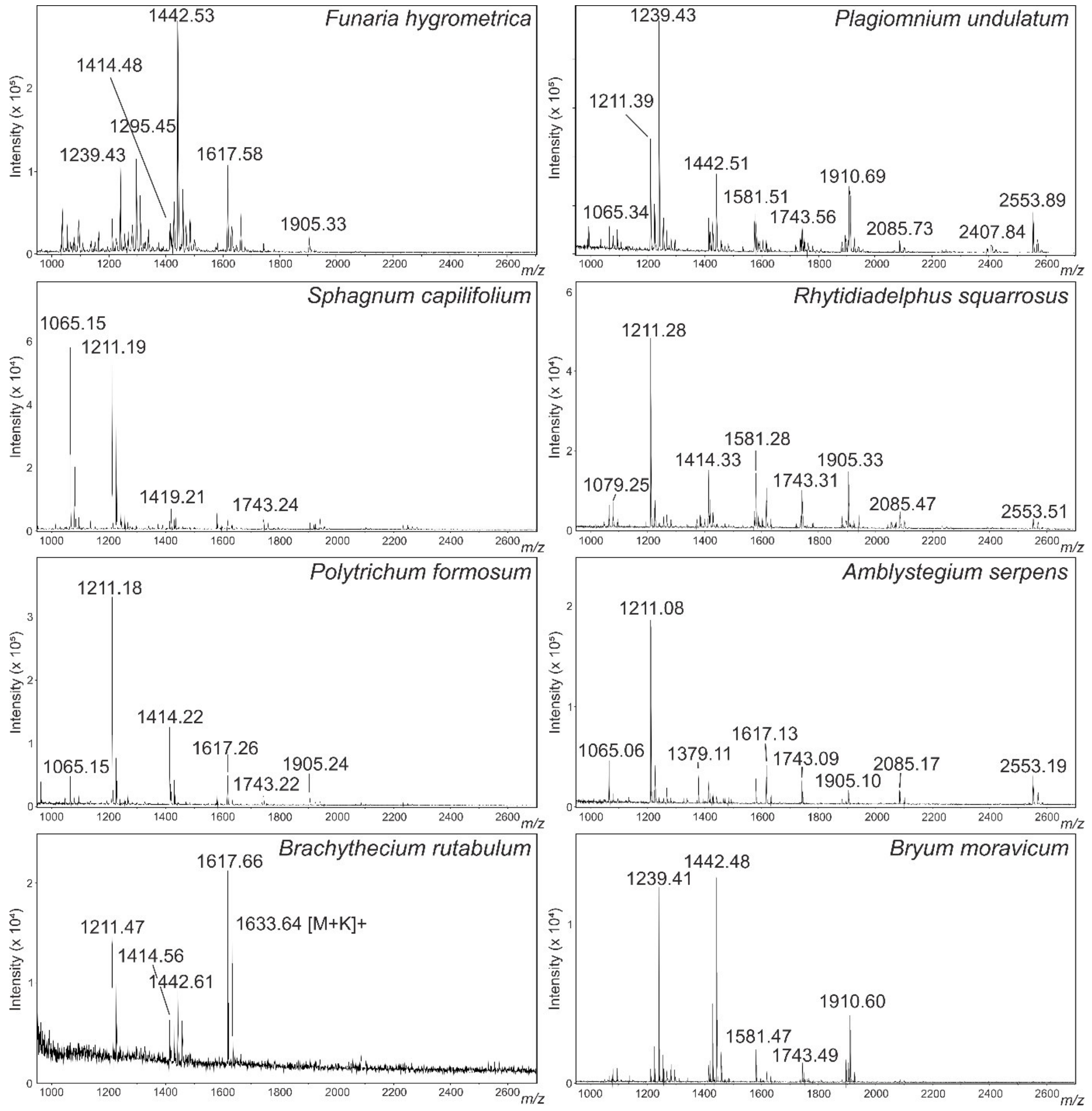

Figure S3B: **MALDI-TOF-MS of different moss species.** Whole glycan pools from the analysed moss species.

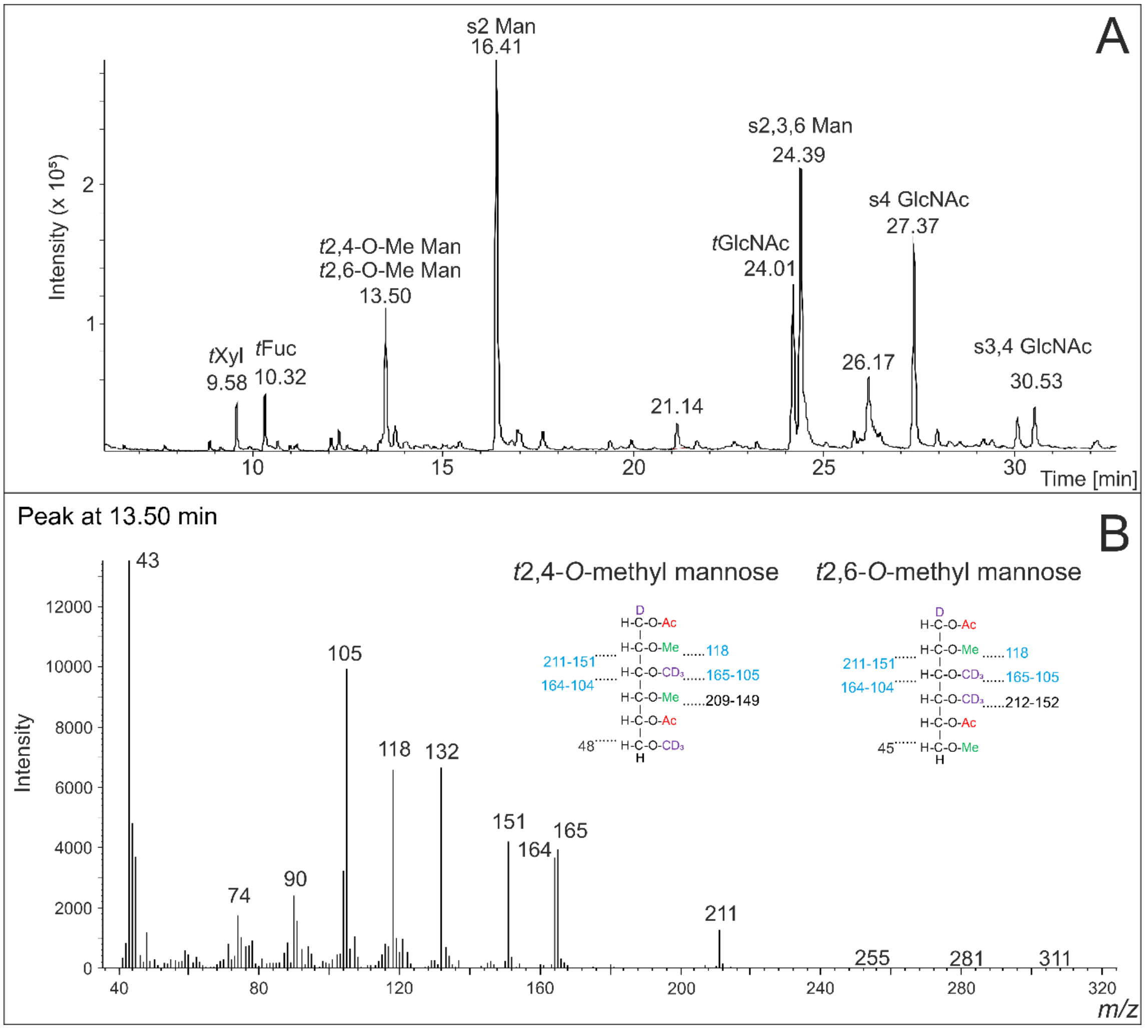

Figure S4A: (**A**): **GC-MS measurements of the HILIC-fractionated main glycan from *Funaria hygrometrica*.** The chromatogram from the first measurement of the sample treated with iodomethane-d3 revealed the doubly methylated mannose in a MGnXF. (**B**): The MS spectrum of this experiment could not clearly distinguish between 2,4-methylated mannose or a 2,6-methylated mannose.

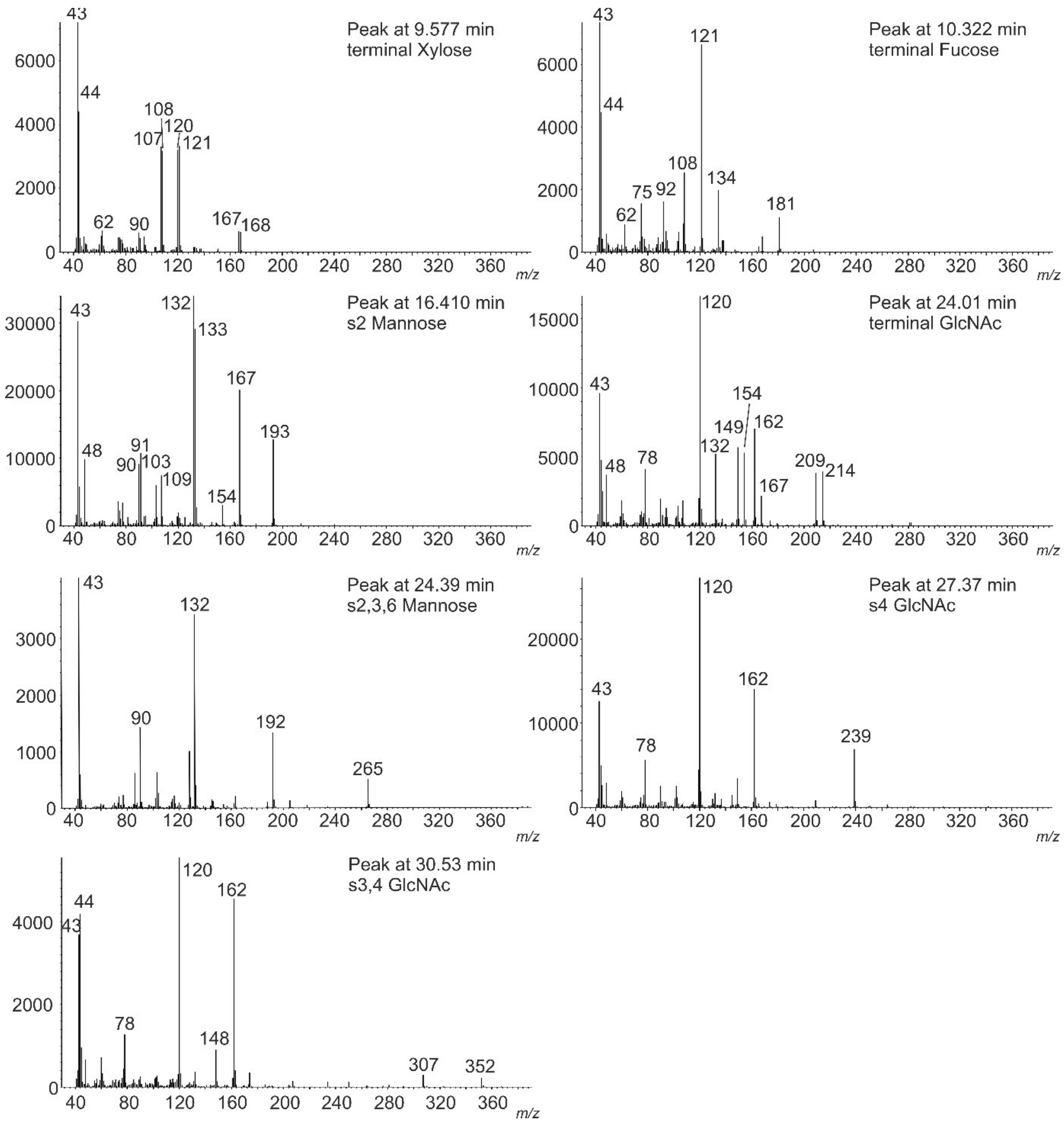

Figure S4B: **GC-MS measurements of the hydrolysed, peracetylated main glycan from *Funaria hygrometrica* treated with iodomethane-d3 before hydrolysis.** The resulting MS-spectra revealed the linkages between the monosaccharides as well as the possible methylation sites.

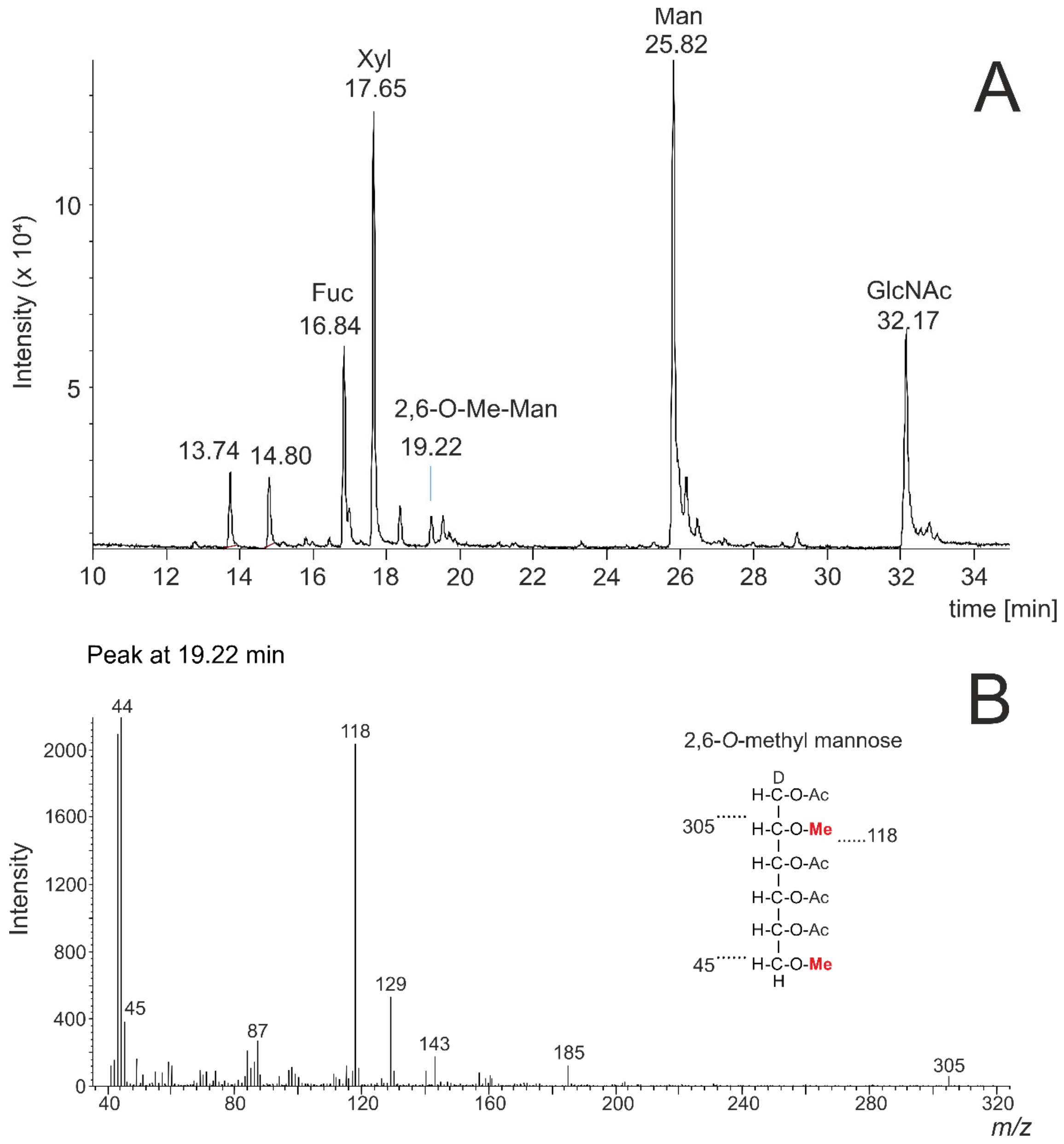

Figure S5A: **Localization of the methyl groups on the mannose of MGnXF of *Funaria hygrometrica*.** (**A**): GM-MS chromatogram of the hydrolysed and peracetylated main glycans from *Funaria hygrometrica* without the treatment of iodomethane-d3. (**B**): MS spectrum of the peak "19.22 min". With these measurements the methylation sites of the mannose were shown.

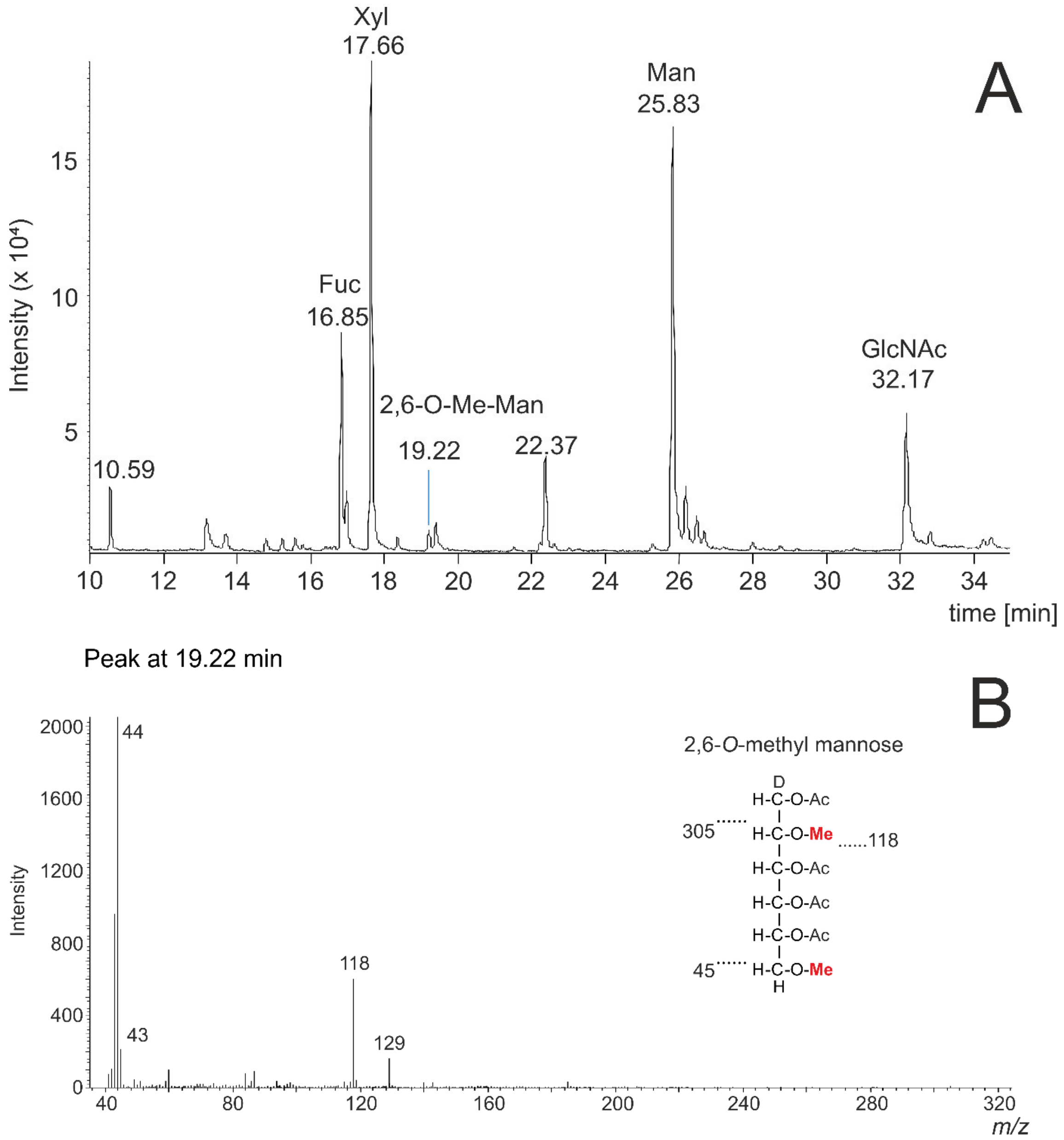

Figure S5B: **Localization of the methyl groups on the mannose of MMXF of *Plagiomnium undulatum*.** (**A**): GM-MS chromatogram of the hydrolyzed and peracetylated main glycans from *Plagiomnium undulatum* without the treatment of iodomethane-d3. (**B**): MS spectrum of the peak "19.22 min". With these measurements the methylation sites of the mannose were shown.

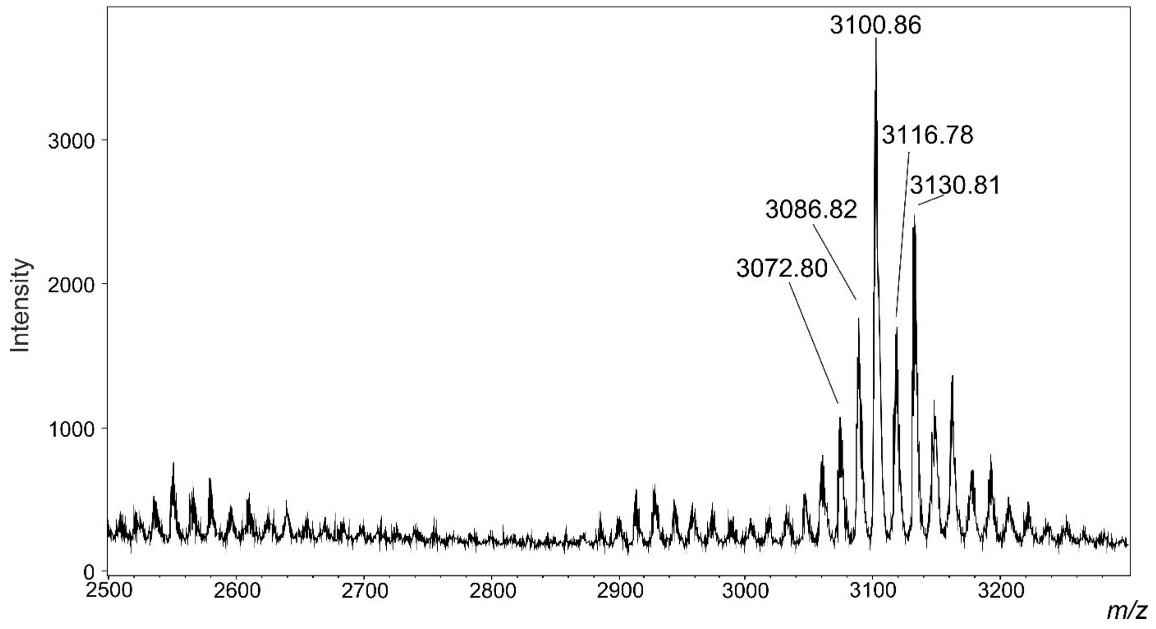

Figure S6: **MALDI-MS spectrum of permethylated 2553 [M+Na]$^+$ of *Plagiomnium undulatum*.** 3100.86 [M+Na]$^+$ represents the permethylated glycan.
